# The Interplay of Orofacial Morphology, Gonial Angle, and Emotional Regulation in Speech and Functional TMJ Impairment and Personalized Approaches

**DOI:** 10.3390/medicina61101886

**Published:** 2025-10-21

**Authors:** Stefan Lucian Burlea, Laura Elisabeta Checheriţă, Ovidiu Stamatin, Diana-Andreea Ilinca, Vasilica Toma, Vlad Proca, Maria Antonela Beldiman, Ana Elena Sîrghe, Georgeta Burlea, Tudor Hamburda, Gabriel Goian, Anamaria Ciubară

**Affiliations:** 1 3rd Dental Medicine Department, Faculty of Dental Medicine, “Grigore T. Popa” University of Medicine and Pharmacy, 700115 Iasi, Romaniaovidiu.stamatin@umfiasi.ro (O.S.);; 2 2nd Dental Medicine Department, Faculty of Dental Medicine, “Grigore T. Popa” University of Medicine and Pharmacy, 700115 Iasi, Romania; 3 1st Dental Medicine Department, Faculty of Dental Medicine, “Grigore T. Popa” University of Medicine and Pharmacy, 700115 Iasi, Romania; 4 Psychology-Speech Therapy Discipline, “Grigore T. Popa” University of Medicine and Pharmacy in Iași, 700115 Iasi, Romania; 5 Prosthetics Department, Faculty of Dental Medicine, “Grigore T. Popa” University of Medicine and Pharmacy, 700115 Iasi, Romania; 6 Phsihiatric Department, Faculty of General Medicine, “Dunarea de jos” University of Medicine and Pharmacy, 700115 Galati, Romania

**Keywords:** dislalia (DIS), dental arch anomalies, orofacial development, gonial angle (GA), ED, FTMJD, pediatric speech disorder (SD), early intervention, interdisciplinary therapy, personalized approaches

## Abstract

*Background and Objectives*: Speech sound disorders, particularly dislalia (DIS), often stem from multifactorial anatomical, functional, and emotional causes during child development. Early identification of risk factors can improve therapy outcomes and prevent long-term communicative and social impairments. This study aimed to assess the relationship between structural (orofacial anomalies, dental arch morphology, and gonial angle (GA)), emotional, and therapeutic variables as predictors of DIS and its subtypes in children aged 5–12 years. *Materials and Methods*: A cross-sectional observational study was conducted on 121 pediatric subjects (58 boys; 63 girls; median age 7.5 years) using clinical examination, standardized speech assessments, emotional-behavioral questionnaires, and radiological imaging(GA measurement). Associations between DIS types, TMJ function, anatomical variation, and therapy outcomes were analyzed using chi-square tests (χ^2^), odds ratios (ORs), and 95% confidence intervals (CIs). *Results*: Cleft-type (OR = 21.43; *p* = 0.003), asymmetrical (OR = 14.66; *p* = 0.004), and crossbite arches (OR = 6.43; *p* = 0.013) significantly predicted DIS. A GA > 130° and <120° trended toward increased speech and motor dysfunction (OR = 4.67; *p* = 0.086). Emotional dysregulation (ED) moderately increased the functional temporomandibular joint dysfunction (FTMJD) risk (OR = 2.26; *p* = 0.060). Early therapy initiation (<7 years) and FTMDJ normalization were consistently associated with superior speech improvement outcomes (OR = 3.10 and 2.40; *p* < 0.01). *Conclusions*: DIS is strongly impacted by structural craniofacial anomalies, particularly cleft-type arches and severe jaw angle deviations. Our findings provide evidence that preliminary personalized approaches and emotional regulation may be beneficial for improving treatment outcomes. These exploratory associations support the rationale for interdisciplinary screening in pediatric populations, but confirmation in multicentric and longitudinal studies is needed.

## 1. Introduction

The stomatognathic system is a dynamic, integrated network of anatomical structures—including the jaws, teeth, temporomandibular joints (TMJs), muscles, lips, palate, and associated soft tissues—that functions harmoniously to enable critical activities such as chewing, swallowing, speaking, and breathing [1,2]. In childhood, its proper development is fundamental to overall health and craniofacial growth. Homeostasis within this system—its ability to self-regulate and maintain optimal function despite ongoing morphological changes and environmental influences—plays a key role in ensuring its long-term integrity [3].

Temporomandibular joint dysfunctions (TMDs) are increasingly recognized in pediatric populations. Children may present with jaw pain, clicking or popping noises, headaches, and restricted mouth opening—manifestations of functional temporomandibular impairments (FTMJDs) [4,5]. Such dysfunctions are often linked to trauma, parafunctional habits such as bruxism, or developmental abnormalities [6]. If not addressed early, FTMJ can disrupt normal jaw growth and function, potentially impacting other components of the stomatognathic system [7].

Morphological changes and occlusal parameters are central to pediatric dentistry. During child development, the size and spatial relationships of the jaws, the contour of the palate, the position of lips and teeth, and the mandibular angle (GA) all evolve. These elements influence the type and severity of occlusal relationships, including malocclusions such as Class I, II, or III patterns [8]. For instance, an increased GA may predispose children to Class II malocclusion, affecting both function and facial aesthetics [9]. Meanwhile, factors like a high-arched palate or improper lip and jaw positioning may complicate both dental and speech development [10].

Social and environmental variables, including gender and residential setting, also play a role in the prevalence and management of stomatognathic disorders. While some studies suggest minor gender differences, the overall prevalence by gender is inconsistent [11]. Urban versus rural residency is associated with disparities in access to orthodontic therapy, which may affect rates of untreated malocclusion [12,13].

A particularly relevant SD associated with stomatognathic anomalies is DIS. DIS is defined as the incorrect production of certain speech sounds, in the absence of neurological or significant sensory impairment. Children with structural anomalies such as malocclusion, a high palate, or abnormal jaw/tongue posture (TP) are more likely to experience articulatory errors—including substitutions, omissions, or distortions of consonants—which potentially lead to academic and social challenges [14]. DIS most commonly affects sibilant and lingual sounds, and while it can occur in both boys and girls, some research notes a slightly higher incidence in males [15].

Orofacial anomalies—such as crossbite, cleft palate, retrognathism, ankyloglossia (tongue-tie), macroglossia, and malocclusion—may disrupt the synchrony of oral-motor movements, alter tongue and lip posture, and directly impact speech intelligibility [16,17]. Among the TMDs, DIS stands out as one of the most frequent speech sound disorders (SSDs) of childhood.

DIS is characterized by persistent substitution, distortion, omission, or addition of phonemes, most commonly affecting sibilants, labials, and liquids (e.g., sigmatism (SIG), betacism (BET), and rhotacism (ROT)) [18]. It can lead to communication difficulties, impaired academic achievement, and reduced social confidence, especially when persisting beyond the typical period of articulatory maturation [19]. DIS encompasses several subtypes:

- SIG—incorrect sibilant sounds, often due to interdental or lateral tongue placement;
- ROT—distortion or substitution of /r/;
- BET—labial errors (misproduction of /b/, /v/, or other labials);
- Palatal or dental DIS linked to structural anomalies such as cleft palate or malocclusion).

Its multifactorial origins include anatomical constraints (arch form, occlusion, tongue mobility, TP), functional deficits (atypical swallowing, reduced oral-motor tone), and behavioral influences, including parafunctional habits and the emotional state [20,21].

Efforts to predict therapeutic outcomes for DIS often focus on the severity of the morphological component, the patient age at intervention, and the type of malocclusion present [22].

However, comprehensive, evidence-based predictive models remain limited, which underscores the need for individualized, multidisciplinary management approaches involving both dental and speech professionals [23,24].

Given these complex and sometimes contradictory data, this study aims to clarify how anatomical (especially dental arch form and GA), functional (TMJ status), emotional, and environmental factors combine to influence the prevalence and therapeutic outcomes of DIS and related orofacial disorders in children. By employing multimodal assessment, validated psychometric tools, and robust statistical analyses, our goal is to provide practical, evidence-based guidance for early risk stratification and effective, targeted prevention and intervention in pediatric stomatognathic care.

The primary aim of this study was to evaluate the influence of anatomical structures, orofacial anomalies, and functional parameters on the prevalence and severity of DIS and FTMJD in children aged 5 to 12 years. Specifically, it sought to assess how variables such as dental arch morphology, GA deviation, and ED are associated with phoneme-specific articulation errors and oral-motor coordination.

This study also aimed to determine whether early interdisciplinary intervention, TMJ normalization, and emotional stability significantly predict improved speech therapy outcomes, thereby identifying key domains for early diagnosis and a multidisciplinary treatment approach in pediatric personalized stomatognathic care.

## 2. Materials and Methods

### 2.1. Study Design:Type: Cross-Sectional Retrospective and Observational Study

Participants: 121 pediatric patients (ages 5–12 years). Gender: 58 boys, 63 girls.

Residence: 63 urban, 58 rural.

For the purposes of this research, data collection was undertaken from 2021 through 2025.

### 2.2. Inclusion/Exclusion Criteria

Inclusion: at least one speech articulation disorder (SD), DIS, complete stomatognathic clinical evaluation, parental consent.

Exclusion: syndromic craniofacial anomalies, diagnosed neurological impairment.

### 2.3. Clinical Assessment Protocol

Intraoral and extraoral inspection (dental arch, tongue, palate, labial competence):

- TMJ palpation, range of motion test, deviation tracking;
- Occlusion type classification (Angle Class I–III, open bite, crossbite);
- Speech evaluation for phoneme-specific DIS types (SIG, BET, ROT, PIT etc.).

Myofunctional assessment (tongue mobility, swallowing pattern, bruxism screening).

### 2.4. Imaging Protocol

- Dental imaging: Panoramic radiographs for GA, tooth alignment, occlusal profile—primarily determined through clinical intraoral examination combined with cephalometric imaging;
- Head and neck imaging: Lateral cephalograms and an adjunctive tool—semi-quantitative and operator-dependent nature. Non-invasive)—radiation-free evaluation; ultrasound for soft tissue symmetry and TMJ orientation. Protocol: Reference points included the lateral condylar margins and mandibular ramus outline, with symmetry judged during standardized mandibular opening and closing movements.

### 2.5. Behavioral and Emotional Evaluation

Behavioral and emotional status was evaluated using parent-reported questionnaires adapted from the Strengths and Difficulties Questionnaire (SDQ), with emphasis on the Emotional Symptoms and Hyperactivity/Inattention domains.

The SDQ is a validated screening tool for psychosocial stress and behavioral difficulties in pediatric populations. To complement this, a structured pediatric checklist was used to identify sleep disturbances and anxiety-related features.

For the purposes of this study, Emotional Alteration (EA) was operationally defined as the presence of at least two positive indicators across these domains (e.g., anxiety traits, irritability, or sleep bruxism).

### 2.6. Data Analysis

Software: SPSS 27, Python 3.8.

Statistics: descriptive statistics, correlation coefficients (Pearson, chi-square), graphical interpretations (bar charts, scatterplots).

Significance threshold: *p* < 0.05.

The normality of the data distribution was assessed using the Shapiro–Wilk test, and the assumptions for parametric testing were met.

Following ANOVA, we applied Bonferroni post-hoc analysis to identify pairwise differences between OSA severity groups across QoL domains. These results have been included in the updated figures, in the specific section.

We conducted multiple linear regression analyses to evaluate the influence of AHI, BMI, age, and gender on each QoL domain presented.

### 2.7. The Ethics Committee of the “Grigore T. Popa”

University of Medicine and Pharmacy in Iaşi granted approval (Approval Code: Nr. 176/17.04.2022). Written informed permission was obtained from each legal guardian or parent.

No therapeutic treatments were used, and the children’s participation was voluntary.

The Declaration of Helsinki’s ethical guidelines were adhered to in this investigation.

## 3. Results

This study included 121 pediatric subjects (mean age: 7.5 years; range 5–12), of whom 58 were boys (47.9%) and 63 were girls (52.1%). The participants were evenly distributed across urban (n = 63) and rural (n = 58) environments. Overall, the distribution of orofacial anomalies, occlusal morphology, and speech disorders (SDs) was analyzed using chi-square (CHI^2^) tests (χ^2^), odds ratios (ORs) with 95% confidence intervals (CIs), probability values (*p*-value *p* < 0.05), and additional descriptive statistics including medians and interquartile ranges.

### 3.1. Orofacial Anomalies and Speech-Related Functional Impact

#### 3.1.1. Frequency and Gender Distribution

Table 1 presents the distribution of functional orofacial anomalies across genders. The median number of orofacial anomalies per child was 1 (IQR: 1–2). No statistically significant trends or differences between boys and girls were observed for any individual condition (all *p* > 0.05), although male patients showed descriptively higher percentages in most categories.

In Table 1, total of 12 orofacial conditions were analyzed by frequency, sex distribution, and relation to potential SDs. While a higher percentage of boys showed each anomaly compared to girls, none of these comparisons reached statistical significance (all *p*-values > 0.05). The OR ranged from 1.34 to 1.72, with all 95% confidence intervals crossing 1.00, indicating that the observed differences could be due to chance:

- LA/DA and macroglossia, both influential in producing SIG and BET, were observed more frequently in boys (12.07% vs. 7.94% and 10.34% vs. 6.35%, respectively), but without statistical significance (*p* = 0.43, OR = 1.60; *p* = 0.37, OR = 1.72);
- These findings suggest that, while male children show numerically higher prevalence, sex alone is not a significant risk factor for individual orofacial dysfunctions (OFD).

None of the conditions showed statistically significant gender association.

While descriptive trends suggest higher male (boys) prevalence in most categories, these differences are not statistically significant. The effect sizes remain clinically moderate (OR range: 1.3–1.72).

Clinical trends suggest male predisposition, possibly due to developmental or skeletal timing differences, although this requires larger studies to confirm.

#### 3.1.2. Dental Arch Anomalies and DIS Risk

Table 2 presents DIS risk relative to dental arch morphology.

Children with asymmetrical, crossbite, or cleft-type dental arches demonstrated significantly increased odds of DIS compared to those with symmetrical arches.

Table 2 presents a powerful anatomical correlation. Children with non-symmetrical arch types—particularly cleft-type, crossbite, and asymmetrical arches—had significantly greater odds of presenting with DIS:

- Cleft-type anomalies presented the highest DIS risk with an OR of 21.43 (95% CI: 2.18–211.0; *p* = 0.003). This extremely high OR marks cleft-type arch formation as the most important anatomical risk for phonological articulation errors observed in this study;
- Asymmetrical arches conferred an OR of 14.66, and crossbites had an OR of 6.43, both of which are statistically significant;
- Symmetrical arches are considered the reference group, with a baseline DIS rate of 31.8%.

Highly significant associations (*p* < 0.01) were observed for all three non-symmetrical arch types.

The ORs confirm a dose-dependent risk progression: symmetrical < crossbite < asymmetrical < cleft-type.

This suggests that craniofacial asymmetry is a primary predictor of DIS types, including ROT and SIG.

Children with cleft-type arches had a 21.4× higher risk of DIS compared to those with symmetrical arches (*p* = 0.003), followed by asymmetrical (OR = 14.66) and crossbite formations (OR = 6.43).

#### 3.1.3. Anatomical Correlations for Dislalia Subtypes

Table 3 demonstrates the main anatomical drivers for each speech sound error, the gender prevalence, and statistical associations for DIS types.

DIS subtypes show important anatomical association but not significant gender differentiation.

The impact of anatomical pathology is most obvious for ROT (cleft, frenulum) and SIG (crossbite, macroglossia), while complex/mixed forms suggest severe or multiple anomalies.

DIS subtypes show strong anatomic associations, but not statistically significant gender effects (all *p* > 0.05).

ROT was most associated with cleft-type palates and a short lingual frenulum—both of which restrict the tongue elevation and positioning required for /r/ sounds. Although it was more common in boys (58%), the difference was not significant (*p* = 0.45).

SIG showed equivalent rates across genders and was predominantly linked to crossbite and macroglossia. These children typically had a poor anterior oral seal and mandibular misalignment leading to distorted /s/ and /z/ sounds.

BET appears in children with LA and retrognathism, primarily affecting bilabials (/b/, /p/) and plosives. There was a slight male trend (OR = 1.33) but no significance (*p* = 0.48).

Mixed DIS mostly occurred in boys (five of six cases). Although the OR = 5.0, the small sample size leads to a wide 95% CI (0.55–45.40) and the *p*-value = 0.26, indicating non-significance.

All DIS types are significantly dictated by anatomical conditions, not child gender.

The strongest associations observed as follows:

- ROT ↔ cleft-type + short frenulum;
- SIG ↔ crossbite + macroglossia;
- BET ↔ LA + retrusion;

While boys had slightly higher percentages across all DIS subtypes, this appears to be a clinical trend, not a statistical fact in this sample.

### 3.2. GA and Speech-Motor Impairment

Table 4 shows distribution of GA ranges and associated oral-motor deficits or FTMJD.

- GA < 120°: Six children, the majority of which were boys (66.7%). DIS: high prevalence (66.67%). ROT: present in 33%, with lower SIG (16.7%) and BET (16.7%). No mixed cases. Motor impairment and FTMJD: 33% had oral-motor impairment; FTMJD seen in two-thirds (66.7%). Children with acute (smaller) angles commonly display ROT and increased oral-motor and TMJ dysfunction, which is possibly linked to mandibular retrusion and reduced oral cavity space;
- GA 120–130° (reference group): 21 children, nearly equally boys and girls. DIS: lower rate (28.6%). The subtypes were well-distributed but less frequent (all <10%). Motor impairment and FTMJD: oral-motor impairment (28.6%) and FTMJD (38.1%) are the lowest among all groups. The normal angle group shows the lowest risk for both DIS and motor impairment, which supports a protective effect of average mandibular morphology for both speech and jaw function;
- GA > 130°: nine children, again more boys (55.6%). Dislalia: strikingly high rate (77.8%). ROT most common (33.3%), followed by SIG (22.2%) and lower mixed/BET. Motor impairment and FTMJD: Highest observed oral-motor impairment (55.6%) and FTMJD (66.7%). Children with an obtuse/large GA (>130°) showed a trend toward higher rates of DIS, oral-motor impairment, and FTMJD. While prevalence patterns suggest possible biomechanical maladaptation, these findings did not reach statistical significance and should be interpreted as preliminary indications.

GA morphometry was analyzed for its relation to both oral-motor impairment and FTMJD:

- Children with greater than 130° GA had increased oral-motor impairments (55.6%) and FTMJD (66.7%), twice as frequent as the reference group (120–130°). This group had an OR of 3.33. However, the 95% CI (0.63–17.47) suggests imprecision due to the small sample size, and the result was not statistically significant (*p* = 0.31);
- Similarly, the GA < 120° children group showed elevated impairment rates (33.3%), but this trend was also non-significant (OR = 1.40; *p* = 0.48). Overall, no statistically significant differences were observed between groups. Nevertheless, children with extreme gonial angles (>130° or <120°) showed clinical trends that were suggestive of increased risk for oral-motor impairment and SD. These results should be interpreted with caution given the limited statistical power, but they point toward possible biomechanical maladaptation as a contributing factor to SIG and ROT.

Jaw biomechanics likely influence articulatory efficiency:

Larger angles may reduce masseter leverage and jaw control.

Acute angles may shorten vertical oral space, affecting tongue tip precision.

The normal angle group (120–130°) consistently exhibited the lowest levels of SD, motor dysfunction, and FTMJD, which supports its use as a baseline reference in morphology-based assessment.

#### 3.2.1. EA and Therapy Timing as Outcome Predictors

To characterize the influence of emotional status and timely intervention on DIS subtypes and outcomes, we analyzed emotional profile data and their relationships with specific phoneme errors.

After administering the SDQ, parent-reported questionnaires, and assessments for children’s emotional status, we obtained the results presented in Table 5.

There was no discernible correlation between SD type and emotional profile (χ^2^ = 1.314, *p* = 0.971).

Although there was no statistical evidence, the anxiousness group had the highest ROT.

The gender balance is preserved across subtypes and emotions, which indicates that emotional state does not influence how DIS is presented.

#### 3.2.2. Emotional Dysregulation, Therapy Outcomes, and DIS Type

Low speech therapy response and emotional problems, particularly anxiety and irritation, were found to frequently co-occur during screening. These findings are summarized in Table 6.

Table 6 investigates therapy outcomes in relation to emotional regulation, therapy timing, and TMJ normalization, quantifying their predictive value for improvement and phoneme resolution.

- Emotional stability and early therapy (<7 years) dramatically increased therapy success (OR up to 3.10, *p* < 0.01);
- Normalization of TMJ function highly improved speech outcomes, especially for structural dislalia (DIS);
- ED nearly doubled the risk for persistent impairment, which emphasizes the need for holistic management encompassing psychological and anatomical domains.

Anxiety and other emotional issues worsened therapy outcomes (OR = 2.26, *p* = 0.060), especially for ROT, BET, and LAM (lambdacism). Early multidisciplinary personalized therapy and TMJ normalization significantly improved success rates.

#### 3.2.3. Correlation Matrix: Summary of Key Predictors and Outcomes

Table 7 presents a correlation matrix which synthesizes the independent and combined impacts of anatomical (arch type, jaw morphology), emotional, and therapeutic variables on DIS occurrence and speech therapy success.

The median age at which therapy was started was 6.0 years (IQR: 5.0–8.0). The two anatomical features that best predicted DIS were crossbite and cleft-type arches (OR up to 21.4; *p* < 0.01). Low ED and early interdisciplinary treatment (before age 7) both markedly enhanced the results (OR = 3.1 and 2.4, respectively; *p* < 0.01). Gender did not significantly affect the prevalence of DIS subtypes or the distribution of anomalies.

There was a synergistic effect of craniofacial structures and emotional well-being on treatment responsiveness. 

The multiplicative impact of emotional and anatomical components in pediatric DIS is highlighted by this matrix. 

Strong risk factors include cleft-type and unequal arches, and early treatment and emotional fortitude yield the best therapeutic results. 

A steady emotional profile and TMJ normalization both increase the effectiveness of therapy. The main outcomes in this sample were not significantly impacted by either gender or environment.

## 4. Discussion

The frequency of major orofacial abnormalities, such as LA/DA, retrognathism, atypical swallowing, palatal cleft, altered dental occlusion, and others, did not significantly differ by gender in our cohort study group of 121 children associated with anatomical predispositions and orofacial conditions. None of the ORs achieved statistical significance, and all were close to or slightly over unity. These findings are consistent with broader population-based cohorts that show little to no variation in childhood orofacial abnormalities by gender. The findings indicating that anatomical risk is mostly gender-independent are supported by the fact that, for example, neither Kulesa-Mrowiecka et al. (2024) nor Mogren et al. (2022) found a significant difference in the prevalence of apraxia between boys and girls with cleft/lip palate disorders [25,26].

Crossbite, cleft-type, and asymmetric dental arch forms were revealed to be strongly predictive of DIS in the section on morphological dental arch anomalies and speech impairments (OR: ~6.4–21; *p* < 0.01). This finding is in line with Farronato, M. et al. (2020) and Rezaei, P. et al.’s (2022) findings that children with malocclusion, clefts, or crossbites had a significantly higher chance of SD [16,27]. Similarly, Assaf et al. found that cleft palate and posterior crossbite were important risk factors for speech distortion [28].

Arch morphology has been mechanistically related to changed TP and phoneme generation by Takayuki Ito (2019) or Shah G. (2020) [29,30]. Particularly in Class II skeletal patterns, abnormal tongue posture—such as pushing or resting low—is linked to thinner, constricted arches and an increased likelihood of malocclusion. Proper TP promotes stable, clear speaking by providing expansive pressure that supports the growth of the arch. Dental arch dimensions and TP are intimately related. The arch establishes the limits for tongue mobility, and tongue position affects the arch’s width and symmetry, all of which are necessary for the best possible speech and occlusal balance. Using somatosensory data and specific muscle activation that are continuously adjusted to each vocal situation, the tongue simultaneously uses dynamically tunable reflexes for real-time stabilization during speech [29].

In the analysis of gonial angle morphometry and TMJ functionality in children, we found that children with an obtuse/large GA (>130° and <120°) showed a trend toward higher rates of DIS, oral-motor impairment, and FTMJD (66.7% vs. 38.1%) and an OR of 3.33 (95% CI: 0.63–17.47) for dysfunction rates. While prevalence patterns suggest possible biomechanical maladaptation, these findings did not reach statistical significance and should be interpreted as preliminary indications. Lee K. et al. (2021) also observed only weak or non-significant links between the gonial angle and masticatory dysfunction in pediatric samples [31]. Shrestha R. et al. (2022) further noted that, while gonial extremes can correlate with malocclusion, they do not causally predict functional TMJ disorders [6,32].

The temporomandibular joint (TMJ) in children, whether functioning or disordered, adjusts dynamically to occlusal alterations, craniofacial growth, and functional demands from speech and mastication interactions. 

Common symptoms of pediatric TMJ problems include joint noises, deviations, or pain, frequently accompanied by developmental abnormalities or parafunctional behaviors. According to epidemiologic surveys, Vallin S., et al., 2024, these illnesses may resolve on their own but may recur when structural risk factors or emotional stress are present [7,33]. Joint noise, orofacial pain, and restricted mouth opening are early clinical indicators that call for an early functional evaluation. According to recent systematic reviews, early diagnosis of TMJ restrictions is crucial for preventing adolescent chronic pain syndromes [8,34]. As our research found, focused TMJ treatment might help individuals articulate speech more clearly. Future longitudinal studies should confirm the potential significance of prompt TMJ examination in pediatric orofacial care, as indicated by this preliminary connection. The significance of prompt TMJ evaluation in pediatric orofacial treatment is highlighted by our discovery that targeted TMJ therapy independently predicts improved speech outcomes.

From ED, TMJ dysfunction, and therapy outcomes, we remarked that:

ED appeared to increase the risk for persistent impairment, although this trend was not statistically significant. These findings point toward the potential value of holistic management that considers both psychological and anatomical domains, but further research is needed.

In our study, TMJ normalization, early interdisciplinary intervention, and emotional regulation were each significantly associated with improved speech therapy outcomes (*p* ≤ 0.002). These results align with prior findings reporting higher TMJ symptomatology in emotionally stressed children in Kilinc, H.E. et al. [9,35] and other systematic reviews reinforcing the association between anxiety and TMJ disorders as part of complex biopsychosocial interactions [10,36,37]. Emotional status was assessed using the Strengths and Difficulties Questionnaire (SDQ) together with a structured checklist for sleep and anxiety-related behaviors, with ED defined as the presence of at least two positive indicators. Although validated and widely applied in pediatric screening, these parent-report tools remain subject to reporting bias. Nevertheless, our findings are consistent with recent validations of the SDQ Emotional Subscale as a reliable screener for anxiety and depression in children [38], and are supported by complementary evidence from the short Mood and Feelings Questionnaire [39] and global analyses of hyperactivity disorders [40]. Taken together, these results suggest that such instruments should be regarded as robust screening, estimative tools rather than diagnostic standards, while their integration into interdisciplinary care remains essential for the early identification of at-risk children.

### 4.1. Stomatognathic System Parameters, Occlusion, and TMJ Evolution

The complex interactions between arch shape, occlusion type, and stomatognathic function in the development of speech and TMJ disorders are confirmed by this study. Even in cases of mixed dentition, symmetrical arch alignment promotes TMJ harmony by supporting tongue function and occlusal stability. Children with clefts, severe crossbite, or occlusal imbalance, particularly those who exhibit parafunctional behaviors such as clenching or mouth breathing, on the other hand, exhibited more severe or persistent TMJD, which is consistent with developmental trajectories as reported in literature by Mehra, S. et al. in 2025 [40] and Shah, G.J. et al. in 2020 [30]. 

There were no discernible gender or rural/urban impacts on orofacial pathology or speech abnormalities. The multicentric survey by Sunderajan, T. et al. (2019) [41] and that by Drechsler, R. (2020) [41,42,43] also showed no difference by gender or place of residence, confirming that anatomical and functional variables are the main drivers of clinical risk across demographics [44,45].

In order to emphasize our comprehension, a factor analysis was conducted using an insight from factor and correlation matrix analysis, which identified three dominating latent constructs:

Structural alignment—including dental arch symmetry, GA, and dental classification;

Functional and parafunctional patterns—such as clenching, oral breathing, and TMJ pain;

ED—encompassing anxiety indicators and behavioral instability.

The literature is increasingly coming to the conclusion that orofacial disorders, particularly those related to the TMJ, are caused by a combination of anatomical, functional, and psychosocial factors. These three domains together explained over 80% of the variation in clinical and functional outcomes. This aligns with the frameworks put forward in recent scoping reviews or research papers such as Bertoli, F.M.P. (2018) [45], Pascu, L. et al. 2025 [46], or Ohrbach, R. et al. (2019) [47] which support integrated diagnostic procedures that go beyond static morphology.

Our study’s hopeful outcome was that children who got early interdisciplinary intervention (before the age of seven) significantly improved their therapy success, especially those whose TMJ function was normalized. Similar results across several therapy models were reported by Black, M.M. et al. (2017) [48] and Hirve et al. (2023), which is consistent with this conclusion [49,50,51].

Emotional control and TMJ normalization both served as independent predictors of therapeutic success in our group (*p* ≤ 0.002), which underscores the complementary roles of psychological and functional modulation in the course of rehabilitation. 

This strengthens the case that pediatric stomatognathic personalized management examinations should adopt an early, comprehensive approach that includes speech articulation profiling, emotional screening, TMJ examination, and occlusal classification [52,53,54,55,56].

Our current study’s results are in good agreement with earlier epidemiological research by Grigorova et al. (2020) [57], who found that 52.7% of 550 preschool-aged children in Skopje, North Macedonia, had phonological articulation abnormalities. This prevalence highlights the consistently high burden of articulation difficulties in early infancy and is consistent with our predicted DIS rate of 54.5% in children aged 5–12. Both investigations show a clinically consistent but statistically insignificant male predominance, with all DIS subtypes affecting boys more commonly.

In particular, Grigorova et al. (2020) [57] found that the most common errors were SIG (24.5% in boys; 20.4% in girls) and ROT (17.8% in boys; 16.5% in girls). This is consistent with our data, which also showed that SIG and ROT were common, especially in children with cross-bite, cleft-type morphology, or short lingual frenulum. Moreover, our longitudinal data confirm that therapy started before the age of seven significantly improves treatment results (OR = 3.10; *p* = 0.001), supporting the importance of early identification and intervention in both trials.

Consequently, the universality of these patterns in pediatric speech-pathology populations is reinforced by the parallels in subtype distribution and therapy response across institutional environments [57].

When started before school age, early childhood education greatly enhances the development and integration of children with speech and language problems (Nilfyr, K. et al., 2025) [58].

Early detection of phonological impairments requires accurate assessments using standardized tools, as shown by Filipova, S. et al. (2023), Braynova, T., et al. (2024) and Mahmutović, E.H. (2018) their protocols provide reliable assessments of expressive language abilities [59,60,61]. Undiagnosed mild hearing loss can cause structural abnormalities like SIG, which can change articulatory and perceptual feedback systems, as noted by Shojaei, E. et al. (2017) [62].

According to Yousif (2018), developmental treatments that are adapted to each developmental stage—from infancy to adolescence—are necessary to address recurrent articulation difficulties [63]. 

Linguistic variety is a significant component of phonological acquisition. Hung, Y.-C. et al. (2022) and Nicolas, S. et al. (2021) stress that anatomical features and language-specific phonemic systems both affect articulation [64,65,66]. Lastly, according to the Royal College of Speech and Language Therapists (2010), both clinically and financially, early and customized speech-language therapy is beneficial in long-term public health models [67,68].

### 4.2. Limitation of Our Study

Despite its comprehensive design, the current study has several limitations:

Sample Size and Representativeness

While the sample size (n = 121) provided adequate statistical power for many analyses, some subgroup comparisons (e.g., extreme GA cases or isolated phoneme disorders) were underpowered, which limited the generalizability of these associations. Moreover, the urban–rural distribution, while balanced numerically, may not fully represent the socioeconomic and ethnolinguistic diversity of the broader pediatric population.

Cross-Sectional and Retrospective Design

This study’s cross-sectional and partially retrospective nature makes it difficult to establish strong causal relationships. While associations between GA, dental arch morphology, emotional factors, and speech outcomes were detected, the directionality and developmental progression of these relationships remain unclear

Subjective Behavioral Assessments

Emotional regulation data relied on parent-reported questionnaires, which may introduce reporting bias or inaccuracies influenced by parental perception, educational level, or cultural interpretations of behavior. Additionally, more objective neuropsychological tools (e.g., executive functioning tests) were not utilized. Therefore, the results are interpreted as screening-level associations, and the absence of formal neuropsychological testing (e.g., executive functioning assessment) is acknowledged as a limitation.

Lack of Longitudinal Follow-Up

The absence of follow-up data prevents analysis of the persistence of DIS or TMJ dysfunction post-intervention. It also limits the ability to assess the predictive validity of GA or ED over time.

Imaging Restriction to 2D Modalities: cephalometry and panoramic radiography were suitable for examining the GA and occlusal form, although 3D imaging tools (e.g., CBCT, MRI) could have provided more precise insights into TMJ anatomy, volumetric changes, and complex spatial relationships.

Only 2D panoramic and cephalometric radiographs were employed, primarily to minimize radiation exposure in the pediatric population, in accordance with recommendations from our Institutional EC. Although these modalities were suitable for assessing GA and general arch morphology, they are limited in precision compared to 3D imaging techniques such as CBCT or MRI.

### 4.3. Future Research

Given the insights and limitations of this study, future investigations should consider the following thematic areas

Longitudinal and Cohort-Based Studies

To confirm predictive factors and track progression over time, longitudinal studies that follow children with varying cephalometric and behavioral profiles will be essential in differentiating transient vs. persistent speech-motor disorders and TMJ dysfunction.

Integration of 3D Imaging and Kinematic Analysis

Advances in 3D craniofacial imaging, real-time ultrasound, and facial kinematics could help analyze morphological-functional correlates of speech production more precisely, especially in borderline or subclinical TMJ dysfunction cases. Future studies should incorporate low-dose CBCT or MRI to provide a more comprehensive evaluation of TMJ morphology, occlusal relationships, and spatial anatomical changes.

Emotion-Neuroplasticity: Neurodevelopmental studies using tools such as fNIRS, EEG, or affective neuropsychology batteries might clarify how ED interferes with motor planning and speech-linguistic integration during critical developmental periods.

## 5. Conclusions

These results should be interpreted as preliminary and hypothesis-generating, highlighting suggestive but not definitive links between orofacial morphology, GA, emotional regulation, and FTMJD in pediatric SSD. The ability to draw robust, generalizable conclusions is restricted by the cross-sectional study design, relatively small sample size, use of 2D imaging, and reliance on subjective parental reporting for emotional measures. Future investigations must employ longitudinal, multicenter approaches with objective neuropsychological testing and advanced imaging to substantiate and clarify these associations. Until such data are available, individualized, interdisciplinary interventions and early screening should be viewed as promising but currently unconfirmed strategies, with statements about early intervention and emotional regulation revised to “potentially advantageous, pending further verification” to reflect this study’s investigative and exploratory character.

## 6. Patents

Patent number 125059 was granted under the provisions of law No. 64/1991, republished in the Official Gazette of Romania, part i, no. 541, dated 8 August 2007. The patent covers a device designed for the stage of learning to read and write.

The inventors and patent holders are Georgeta Burlea, Anamaria Burlea, and Ștefan Lucian Burlea, all from Iași, Romania.

This patent was officially issued on 30 August 2013, in Bucharest, Romania.

## Figures and Tables

**Table 1:** Orofacial conditions by gender and association with speech dysfunctions (boy—B; girl—G).

Orofacial Condition	Total N (%)	B n (%)	G n (%)	Chi^2^	*p*-Value	OR (B/G)	95% CI	Primary Speech Impact
Labial Apraxia/Dyspraxia (LA/DA)	12 (4.96%)	7 (12.07)	5 (7.94)	0.62	0.43	1.60	0.47–5.58	PIT, BET
Retrognathism	9 (3.72%)	5 (8.62)	4 (6.35)	0.24	0.62	1.39	0.37–5.29	Jaw retrusion, articulation limit
Atypical Swallowing	9 (3.72%)	5 (8.62)	4 (6.35)	0.24	0.62	1.39	0.37–5.29	Tongue thrusting, imprecise air control
Palatal Cleft	9 (3.72%)	5 (8.62)	4 (6.35)	0.24	0.62	1.39	0.37–5.29	Hypernasality, disarticulation
Altered Dental Occlusion	10 (4.13%)	6 (10.34)	4 (6.35)	0.80	0.37	1.72	0.48–6.23	Distorted resonance
TMJ Ankylosis	11 (4.55%)	6 (10.34)	5 (7.94)	0.28	0.60	1.34	0.41–4.46	Restricted mandibular articulation
Short Lingual Frenulum	10 (4.13%)	6 (10.34)	4 (6.35)	0.80	0.37	1.72	0.48–6.23	ROT /R/ emission problems
Lingual Hypotonia	9 (3.72%)	5 (8.62)	4 (6.35)	0.24	0.62	1.39	0.37–5.29	Phoneme instability
Geographic Tongue	10 (4.13%)	6 (10.34)	4 (6.35)	0.80	0.37	1.72	0.48–6.23	Distorted sensory feedback
Lip Occlusion Deficiency	11 (4.55%)	6 (10.34)	5 (7.94)	0.28	0.60	1.34	0.41–4.46	Bilabial instability
Macroglossia	10 (4.13%)	6 (10.34)	4 (6.35)	0.80	0.37	1.72	0.48–6.23	SIG
Labial Cleft	10 (4.13%)	6 (10.34)	4 (6.35)	0.80	0.37	1.72	0.48–6.23	Resonance, lip seal reduction

**Table 2:** Dental arch type and DIS association.

Arch Type	Total N	B, n (%)	G, n (%)	DIS, n (%)	No DIS, n N (%)	Chi^2^	*p*-Value	OR (Vs. Symm.)	95% CI	ROT, N (%)	SIG, N (%)	BET, N (%)	Mixed N (%)
Symmetrical	22	11 (50.00)	11 (50.00)	7 (31.82)	15 (68.18)	-	-	Reference	-	2 (28.57)	3 (42.86)	1 (14.29)	1 (14.29)
Crossbite	16	9 (56.25)	Arch Type	12 (75.00)	4 (25.00)	6.12	0.013	6.43	1.63–25.36	4 (33.33)	5 (41.67)	2 (16.67)	1 (8.33)
Cleft-type	11	6 (54.55)	5 (45.45)	10 (90.91)	1 (9.09)	8.87	0.003	21.43	2.18–211.0	6 (60.00)	2 (20.00)	1 (10.00)	1 (10.00)
Asymmetrical	13	7 (53.85)	6 (46.15)	11 (84.62)	2 (15.38)	8.13	0.004	14.66	2.34–92.00	5 (45.45)	3 (27.27)	2 (18.18)	1 (9.09)

**Table 3:** Phoneme-specific DIS, anatomical correlates, and gender impact.

DIS Type	B, n (%)	G, n (%)	Total n (%)	Main Associations	Chi^2^	*p*-Value	OR (B/G)	95% CI	Clinical Note
ROT	11 (57.9)	8 (42.1)	19 (28.8)	Cleft palate, short frenulum	0.57	0.45	1.33	0.61–2.91	Restricted tip mobility, resonance
SIG	8 (53.3)	7 (46.7)	15 (22.7)	Crossbite, macroglossia, FTMJD	0.01	0.94	1.03	0.43–2.46	Anterior instability, large tongue
BET	9 (56.3)	7 (43.7)	16 (24.2)	Labial apraxia (LA), retrusion	0.50	0.48	1.33	0.56–3.12	Bilabial/labiodental misarticulation
Mixed	5 (83.3)	1 (16.7)	6 (9.1)	Cleft, arch asymmetry	1.26	0.26	5.00	0.55–45.4	Multiple errors/comorbidities

**Table 4:** GA and motor dysfunction.

GA Range	n,	B, n (%)	G, n (%)	DIS n (%)	ROT	SIG	BET	Mixed	Motor Impairment n (%)	FTMJD n (%)	OR (vs. Ref)	95% CI	Chi^2^ Pearson	*p*-Value
<120°	6	4 (66.7)	2 (33.3)	4 (66.7)	2	1	1	0	2 (33.3)	4 (66.7)	2.62	0.41–16.54	0.36	0.55
120–130°	21	11 (52.4)	10 (47.6)	6 (28.6)	2	2	1	1	6 (28.6)	8 (38.1)	Reference	-	Ref.	-
>130°	9	5 (55.6)	4 (44.4)	7 (77.8)	3	2	1	1	5 (55.6)	6 (66.7)	4.67	0.75–29.09	2.94	0.086

**Table 5:** Emotional status and its influence on DIS.

Emotional Type	Rotacism	Betacism	Sigmatism	Mixed	Rotacism %	Betacism %	Sigmatism %	Mixed %	Chi^2^	Degrees of Freedom	*p*-Value	OR (Rotacism: Anxiety vs. Others)	95% Lower	95% Upper
Anxiety	10	9	7	2	35.7	32.1	25.0	7.1	1.314	6	0.971	1.173	0.387	3.550
Irritability	6	5	5	3	31.6	26.3	26.3	15.8	1.314	6	0.971	1.173	0.387	3.550
Other	3	2	3	1	33.3	22.2	33.3	11.1	1.314	6	0.971	1.173	0.387	3.550

**Table 6:** Therapy outcomes: emotional, temporal, and anatomical predictors.

Predictor	Success (%)	χ^2^	*p*-Value	OR	95% CI	DIS Key
ED (TMJD)	37.0	3.55	0.060	2.26	0.96–5.31	ROT, BET,
Interdisciplinary Therapy < 7 y	84.0	11.29	0.001	3.10	1.90–5.00	All DIS types
TMJ Normalization Achieved	79.5	10.01	0.001	2.40	1.50–3.80	ROT, BET, SIG
Low ED	70.0	9.45	0.002	2.40	1.50–3.80	Stronger recovery

**Table 7:** Correlation matrix: predictors of DIS and therapy results.

Predictor	Outcome	χ^2^	*p*-Value	OR	95% CI	Significance	Interpretation
Crossbite Arch	DIS	6.12	0.013	6.43	1.63–25.36	Significant	Structural risk for sigmatism
Cleft-Type Arch	DIS	8.87	0.003	21.43	2.18–211.0	Highly significant	Highest risk for rotacism
Asymmetrical Arch	DIS	8.13	0.004	14.66	2.34–92.00	Highly significant	Severe phoneme errors
GA > 130°	SD	2.94	0.086	4.67	0.75–29.09	Near significant	Motor disadvantage
ED	FTMJD	3.55	0.060	2.26	0.96–5.31	Near significant	Psychological effect
Early Therapy <7 y	Therapy Success	11.29	0.001	3.10	1.90–5.00	Very significant	Tripled success rate
TMJ Normalization	Therapy Success	10.01	0.001	2.40	1.50–3.80	Significant	Strong outcome boost
Low Emotional Reactivity	Therapy Success	9.45	0.002	2.40	1.50–3.80	Significant	Consistent positive effect

## Data Availability

The raw data supporting the conclusions of this article will be made available by the authors on request.

## Abbreviations

The following abbreviations are used in this manuscript:

TMJ	Temporomandibular Joint
FTMJD	Functional Temporomandibular Joint Disturbance (children)
TMJD	Temporomandibular Joint Dysfunction
SS	Stomatognathic System
GA	Gonial Angle
SD	Speech Disorder
DIS	Dislalia
MG	Macroglossia
AG	Ankyloglossia (Short Lingual Frenulum)
LA/LD	Labial Apraxia/Dyspraxia
ED	Emotional Dysregulation
ODI	Orofacial Dysfunction Index
MFA	Myofunctional Assessment
CSOS	Cross-Sectional Observational Study
OR	Odds Ratio
CI	Confidence Interval
SPSS	Statistical Package for the Social Sciences
EC	Ethics Committee
IC	Informed Consent
SIG	Sigmatism
ROT	Rhotacism
PIT	Pitacism
LAM	Lambdacism
BET	Betacism
SSD	Speech Sound Disorders (SSD)
(χ^2^) Chi^2^	Chi Pearson
*p*-value	Semnificatie Statistica
ODD(OR)	Odds Ratio
